# A Physical-Layer Security Cooperative Framework for Mitigating Interference and Eavesdropping Attacks in Internet of Things Environments

**DOI:** 10.3390/s24165171

**Published:** 2024-08-10

**Authors:** Abdallah Farraj, Eman Hammad

**Affiliations:** 1 Department of Electrical Engineering, Texas A&M University-Texarkana/RELLIS Campus, Bryan, TX 77807, USA; 2 Engineering Technology and Industrial Distribution Department, Texas A&M University, College Station, TX 77843, USA; eman.hammad@tamu.edu

**Keywords:** Internet of Things (IoT), physical-layer security (PLS), wireless communication, information availability, information confidentiality, interference attacks, eavesdropping, cooperative transmission strategy

## Abstract

Intentional electromagnetic interference attacks (e.g., jamming) against wireless connected devices such as the Internet of Things (IoT) remain a serious challenge, especially as such attacks evolve in complexity. Similarly, eavesdropping on wireless communication channels persists as an inherent vulnerability that is often exploited by adversaries. This article investigates a novel approach to enhancing information security for IoT systems via collaborative strategies that can effectively mitigate attacks targeting availability via interference and confidentiality via eavesdropping. We examine the proposed approach for two use cases. First, we consider an IoT device that experiences an interference attack, causing wireless channel outages and hindering access to transmitted IoT data. A physical-layer-based security (PLS) transmission strategy is proposed in this article to maintain target levels of information availability for devices targeted by adversarial interference. In the proposed strategy, select IoT devices leverage a cooperative transmission approach to mitigate the IoT signal outages under active interference attacks. Second, we consider the case of information confidentiality for IoT devices as they communicate over wireless channels with possible eavesdroppers. In this case, we propose a collaborative transmission strategy where IoT devices create a signal outage for the eavesdropper, preventing it from decoding the signal of the targeted devices. The analytical and numerical results of this article illustrate the effectiveness of the proposed transmission strategy in achieving desired IoT security levels with respect to availability and confidentiality for both use cases.

## 1. Introduction

The daily activities of modern living have become more integrated and, in many cases, reliant on technology. This holds true on the consumer level, as well as industrial and enterprise levels. Technologies that provide versatility and connectivity and enable efficient operations with simpler user experience have become prevalent. One of the most dominant technologies in this context are devices that can sense and/or actuate and control some physical quantity, are connected to the Internet, and can communicate with users or other devices. Such devices have become known as Internet of Things (IoT) devices. IoT devices can be loosely categorized into consumer and industrial general types, with predictions expecting the number of connected IoT devices globally to exceed 32 billion by 2030 [1]. IoT expands a large spectrum of technologies from drones, robots, connected vehicles, health devices, controllers, grid electric transformers, and many other industries. IoT devices have existed since the early days of the Internet and have since become an increasingly fascinating manifestation of technological development.

Industrial use cases across many domains extensively utilize IoT to perform sensing and actuation tasks with minimal human intervention [2], thus supporting higher levels of automation and autonomy. Hence, cybersecurity and resilience become critical, specifically in ensuring the integrity, confidentiality, and availability of the IoT devices and communication connectivity to the devices. While IoT devices vary largely in capabilities and the nature of available computational resources, the general trend in the industry optimizes on-device resources such as processing, memory, storage, energy usage, and cost based on functionality and purpose. This often has resulted in IoT technologies suffering from serious security flaws and gaps. In fact, several major cybersecurity attacks during the past few years have leveraged IoT devices as part of the attack kill chain [3,4]. Most recently, many efforts have focused on improving IoT built-in security.

Cybersecurity encompasses technologies and practices to safeguard information’s availability, integrity, and confidentiality. Traditional cybersecurity measures primarily focus on preventing the unauthorized access, disruption, and modification of information. The evolution of such controls was historically based on a special class of technologies (Information Technologies or IT) used in information systems within typical computer networks. Traditional cybersecurity defenses and controls, such as access control, key management, and encryption schemes, often prove impractical for ecosystems with limited storage, processing, and transmission capabilities [5,6,7]. Security priorities in an IoT system rely heavily on the nature of the system, whereas, in delay-sensitive critical infrastructures, availability and integrity are of the highest priority. In other IoT environments, such as health monitoring, confidentiality may be of higher priority. In critical control operations and industrial processes, measures for the confidentiality of information prevent unauthorized access to sensor measurements by an illegitimate eavesdropper, thus avoiding the disclosure of the industrial process’s critical information. Data theft in wireless IoT networks raises concerns related to violations of privacy, infringements of intellectual property, and reverse engineering of system settings.

To fully capitalize on the benefits of IoT ecosystems, it is crucial to apply robust security controls [8,9,10,11,12]. Inadequate security and negligence of proper risk understanding and management may cause significant damage from adversaries, particularly when IoT is part of critical industrial control systems [7,13]. Ensuring information integrity and availability becomes paramount in such environments. Information availability guarantees that controllers receive timely access to IoT-transmitted data as needed. Similarly, information confidentiality measures ensure that only devices allowed to read the information are able to do so.

IoT systems are widely employed in various industries and mostly utilize a form of wireless communication for connectivity. Using wireless communication technologies can support scalability in large-scale IoT systems’ deployments and operations. Machine-to-machine communication links (e.g., Zigbee, LoRa, Bluetooth) often prove to be useful for large-scale deployments [1,14,15,16]. Modern wireless technologies, such as spectrum-sharing communication systems, present new opportunities to enable IoT connectivity [17]. This is particularly interesting in newer generations of cellular communication, such as 6G, where massive machine-type communication continues to be a key driver. Due to the shared nature of the communication channel, wireless IoT networks face critical challenges in ensuring information security [18]. The complexity of emerging security threats targeting IoT devices further exacerbates the issue, especially in resource-constrained IoT systems. Incidents like the Mirai attack have highlighted the vulnerability of IoT systems to cyber attacks [7,18,19].

The dominant use of wireless communication channels in IoT environments cast them as attractive targets for threat vectors that exploit the inherent vulnerabilities in such channels’ physical and data layers. For example, in attacks that target availability, an adversary may intentionally interfere with and degrade wireless communication channels. Such attacks may disrupt industrial control system operations, raising concerns related to health, safety, and quality. Similarly, an adversary who has access to the wireless communication medium may sniff the spectrum to reverse engineer transmitted information.

This work acknowledges current IoT security challenges, particularly in resource-constrained devices, to address IoT interference and eavesdropping attacks. In this article, we present an alternative approach to security at the physical layer, focusing on two use cases with different security objectives. We motivate the physical-layer security (PLS) approach as a complementary approach to other network and application layer mechanisms. Due to the challenges in securing IoT systems and the inherent computational limitations of the devices, PLS methods are becoming more popular [5,6,20,21]. A major benefit of PLS approaches for IoT environments lies in their ability to provide enhanced security within the constrained resources of the IoT devices as we illustrate in this article with the proposed strategies. Other security controls on the network and application layers are often limited due to restricted device resources.

First, we consider the challenge of interference attacks, where we investigate a scenario where an IoT device transmits its sensor data to a receiver unit through a wireless channel that is subjected to an intentional interference attack by an *adversary*. The malicious interference negatively affects the legitimate IoT’s received signal, which results in channel outages that impede timely access to IoT data at the receiver unit, thereby disrupting the availability of IoT data. In the IoT system under investigation, the legitimate device can coordinate its transmission with other IoT devices in the ecosystem to mitigate the negative impacts of the interference attack conducted by the adversary. One objective of the proposed security approach is to limit the average outage probability of the legitimate device’s signal to an acceptable threshold during the interference attack. The approach employed in this work focuses on employing a spectrum-sharing cognitive communication framework [22] to address information availability at the physical layer. Cooperative communications between devices in the IoT ecosystem are employed to enhance the quality of service (QoS) of the received signal during the interference attack.

Second, we consider a setup with several IoT devices utilizing a wireless channel to communicate their sensor measurements. A set of the IoT devices, called primary devices, require higher signal quality guarantees at the receiver compared with the the rest of the devices (called secondary devices), which have lower transmission priority. The primary and secondary IoT devices may use different receiving units. Additionally, there is an illegitimate device, referred to as the eavesdropper, attempting to decode the primary device’s transmission. A coordinated transmission strategy by secondary IoT devices is developed in this article to ensure the information confidentiality of the primary device’s signal in the presence of the eavesdropper.

In the remaining parts of the articles, we discuss security for IoT systems in Section 2, and we discuss the proposed solutions for interference attacks in Section 3 and for eavesdropping in Section 4. Simulation results illustrating the performance of the proposed solutions are shown and discussed in Section 5. Conclusions and future work are presented in Section 6.

## 2. Background and Motivation

Recently, security strategies originally developed for sensor networks have been extended to IoT environments due to their similarities [5,16,23,24,25,26,27,28,29]. However, the widespread deployment of IoT devices, coupled with their unique computational capabilities and energy efficiency, presents challenges for existing security approaches. For instance, security schemes relying on compressive sensing, probabilistic ciphering, and channel state information scalability suffer as the number of devices increases. Additionally, computationally complex schemes like compressive sensing are impractical for resource-limited IoT devices [2,5]. Moreover, the sheer number of IoT devices and the complexity of interconnected systems make it more challenging to identify and address security vulnerabilities.

Physical-layer security leverages wave propagation and transmitter/receiver designs and offers an approach to information security by enabling secure communication over wireless channels [2,5,26,30,31]. In the context of IoT systems, PLS approaches have the capability to overcome some of the constraints of conventional cybersecurity solutions and offer extra layers of protection against cyber attacks [20,21]. It can make eavesdropping and disrupting IoT communications more difficult for attackers without transmitting additional information.

A review of physical-layer security approaches for achieving information security in wireless channels is provided in [5,32]. The challenges and opportunities of using PLS in IoT systems are discussed in surveys such as [31,33,34,35]. Several PLS techniques can be employed in IoT systems, including beamforming to direct signals toward intended receivers and away from eavesdroppers as well as the use of artificial noise to hinder eavesdroppers in decoding transmitted signals. Other existing PLS methods include operating within the secrecy capacity, exploiting channel signatures, using spectrum spreading techniques, and node cooperation to degrade the eavesdropper’s communication channel [36]. Additional results on PLS security are summarized in [6].

The work in [37] investigated security solutions for heterogeneous IoT and multi-access mobile edge computing (MA-MEC) in smart cities, focusing on physical-layer security technologies like secure wiretap coding, resource allocation, signal processing, and multi-node cooperation to address emerging security threats. The researchers in [38] proposed a Gaussian-tag-embedded physical-layer authentication scheme for IoT security, using a weighted fractional Fourier transform to verify signal authenticity, and they conducted security analysis and experiments to demonstrate the scheme’s robustness against spoofing and replay attacks. The study in [39] explored a secure wireless communication scenario in IoT for protecting data collection from detection and eavesdropping attacks. The work in [40] studied secure beamforming design in a two-way cognitive radio IoT network with simultaneous wireless information and power transfer with the aim to maximize the secrecy sum rate for primary users by designing beamforming solutions and optimization algorithms to balance complexity and performance.

Studies have examined the average secrecy capacities of wireless multi-user networks against passive or active eavesdroppers [41]. Physical-layer security approaches for wireless sensor networks include distributed co-phasing-based transmissions [26] and energy-efficient solutions for securing downlink IoT connections through interference exploitation [6]. A unified framework for various physical-layer security systems has been proposed [42]. In [20], physical-layer security measures for an IoT environment under jamming signals are discussed, utilizing a game-theoretic formulation for distributed IoT channel access. However, scaling this game-theoretic approach becomes challenging as the number of IoT devices increases due to transmission collisions and retransmissions.

The proposed solutions for interference and eavesdropping attacks in this article are innovative as they do not waste resources, provide opportunities for IoT cooperation, complement other security measures that are in place, strengthen defense-in-depth strategy, and quantify a measure of information availability and confidentiality using outage probability. The proposed algorithms use a round-robin approach to include secondary IoT devices, providing a chance to communicate over the channel for all devices and leading to more fairness in the IoT network. The algorithms also include a degree of flexibility through setting the value of a cooperation factor. It is important to note here that the proposed cooperative transmission strategy for interference attacks requires accurate estimates of the adversary channel gains, which is feasible using channel estimation techniques for active interfering agents.

In the following, we discuss the proposed PLS solutions for IoTs under interference attack in Section 3 and for eavesdropping attacks in Section 4. The theoretical framework and the cooperative transmission strategies that enable the IoTs to respond to the cyber attacks will be developed for both use cases.

## 3. PLS for Interference Attacks Defense

Consider a communication system consisting of multiple IoT devices that need to transmit data using a wireless channel. Within this ecosystem, certain devices, referred to as primary IoT devices, require higher information availability guarantees at their respective receivers compared to others, known as secondary IoT devices. It should be noted that primary and secondary IoT devices may have different receiver units. In this scenario, an adversary specifically targets the data transmission of a primary IoT device by conducting interference attacks that jam its receiver unit. To address this challenge, a spectrum-sharing cognitive communication paradigm is utilized [43]. Secondary IoT devices can concurrently transmit over the shared channel along with the primary IoT device to ensure a target level of signal quality for the primary device. The primary outage probability is considered as the QoS metric in this setup.

To utilize the channel, the secondary IoT device cooperates with the primary device by allocating a portion of its power to relay the primary device’s signal and using the remaining power to transmit its own data. Consequently, the simultaneous transmission of signals introduces additional interference at the intended receiver. However, the QoS of the received signal can be improved through cooperative communication from the secondary IoT devices in the system. This cooperative communication approach allows the primary IoT device to achieve a certain measure of information availability while under interference attacks by the adversary.

### 3.1. System Model

**Figure 1:**
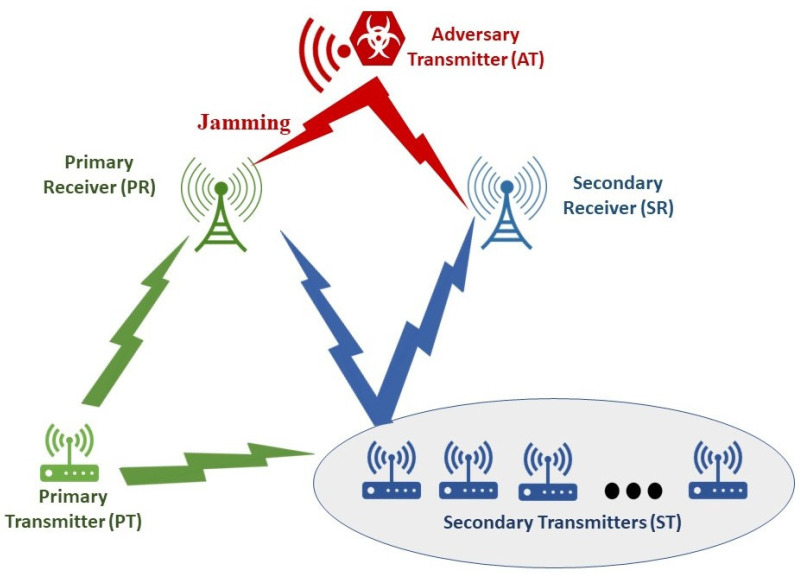
Interference attacks problem setup.

Consider the spectrum-sharing uplink communication environment depicted in Figure 1. This setup includes a legitimate primary IoT device that intends to transmit its data (for example, sensor readings) to a primary receiver unit (PR). Also, the wireless communication environment includes other secondary devices (collectively referred to as ST) that aim to transmit their information to a secondary receiver unit (SR). In this communication system, the PR and SR can simultaneously transmit over the shared wireless channel. Additionally, the communication system includes an adversary device (referred to as AT) that attacks the data transmission of the PT by causing an interference at the PR. In a similar way, the adversary’s transmission introduces additional interference at the SR as well. In addition, the secondary transmission by the ST causes interference at the PR. In a similar fashion, the primary transmission by the PT leads to additional interference at the secondary receiver SR.

Furthermore, the PT utilizes the secondary transmission by the cooperative ST to alter the composition and characteristics of its received signals at the PR, with the goal of limiting the average value of the outage probability of the primary signal at the PR in order to achieve certain degree of information availability during the AT’s interference attack. Throughout the time duration of interest, the PR transmits its data at a rate of $R_{p}$ with a power of $P_{p}$. Each transmission interval involves the selection of a secondary device to communicate over the shared channel with a power of $P_{s}$ and a rate of $R_{s}$. In addition, the adversary user causes interference utilizing a transmission power of $P_{a}$. Finally, the PR and SR experience additive white Gaussian noise (AWGN) signals with zero mean and a variance of $\sigma_{p}^{2}$ and $\sigma_{s}^{2}$, respectively.

**Figure 2:**
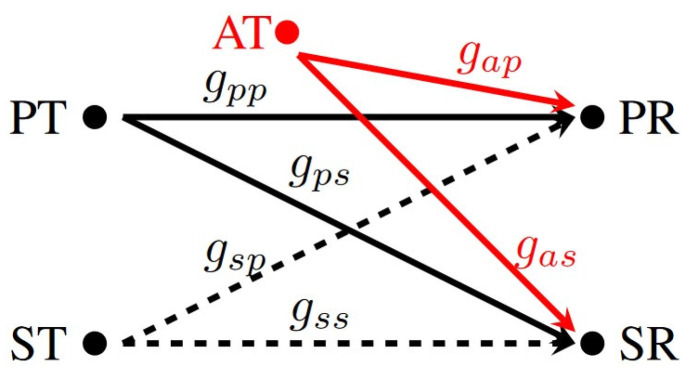
Interference attacks problem model.

The wireless channels between the different IoT devices and receiver units in this environment undergo independent and identically distributed (i.i.d.) Rayleigh block fading. Figure 2 illustrates the power gains of the channels between the PT and PR and the PT and SR as $g_{pp}$ and $g_{ps}$, respectively, with average values of $\lambda_{pp}$ and $\lambda_{ps}$. Likewise, the power gains of the channels between the AT and PR and the AT and SR are termed as $g_{ap}$ and $g_{as}$, respectively, with average values of $\lambda_{ap}$ and $\lambda_{as}$. Finally, the power gains of the channels between the ST and PR and the ST and SR are represented by $g_{sp}$ and $g_{ss}$, respectively, with average values of $\lambda_{sp}$ and $\lambda_{ss}$. These different λ values capture pertinent characteristics of the communication environment, such as propagation distance between the transmitter and receiver units, path loss, shadowing, and the general fading state of the channel.

### 3.2. Cooperation Model

To mitigate the impact of the interference signal injected by the adversary unit and facilitate cooperation with the primary IoT device, the secondary device allocates a portion of its transmission power ($P_{s}$) for relaying the PT’s data. In this communication environment, the following assumptions are made:

1. The PT and ST are relatively close to each other so that the propagation time between the PT and ST is insignificant compared to that between the PT and PR.
2. The ST possesses accurate retransmission capability for PT’s data.
3. The ST dedicates a fraction $\alpha P_{s}$ of its transmission power to cooperate with the PT, and the remaining fraction $\left(1-\alpha \right)P_{s}$ is used for transmitting ST’s own coded signal.

Here, α represents the cooperation factor, satisfying the condition 0≤α<1. Although we realize that the first two assumptions might not be very practical at all times, nevertheless, they provide us with a direct way to derive the following mathematical terms and keep the developed expressions traceable.

Let $\gamma_{p}$ represent the signal-to-interference plus noise ratio (SINR) of the PT’s signal that is received at the PR, and let $\gamma_{s}$ denote the ST’s signal SINR that is received at the SR. Given the concurrent transmissions between the different IoT devices, $\gamma_{p}$ and $\gamma_{s}$ can be expressed as

(1) $$\begin{array}{cc} \gamma_{p} & =\frac{P_{p}g_{pp}+\alpha P_{s}g_{sp}}{\left(1-\alpha \right)P_{s}g_{sp}+P_{a}g_{ap}+\sigma_{p}^{2}}.\\ \gamma_{s} & =\frac{\left(1-\alpha \right)P_{s}g_{ss}}{P_{p}g_{ps}+P_{a}g_{as}+\sigma_{s}^{2}}. \end{array}$$

For the case of Rayleigh fading in the channel, the cumulative distribution function (CDF) of $g_{pp}$ can be written as

(2) $$F_{g_{pp}}\left(x\right)=\left(1-\exp\left(-\frac{x}{\lambda_{pp}}\right)\right)u\left(x\right)$$

where u(·) denotes the unit step function. Similar formulas can be found for the other channel gains in this environment.

The expression for $\gamma_{p}$ can be expanded into $\gamma_{p}=\gamma_{p_{1}}+\gamma_{p_{2}}$, where

(3) $$\begin{array}{cc} \gamma_{p_{1}} & =\frac{P_{p}g_{pp}}{\left(1-\alpha \right)P_{s}g_{sp}+P_{a}g_{ap}+\sigma_{p}^{2}}\\ \gamma_{p_{2}} & =\frac{\alpha P_{s}g_{sp}}{\left(1-\alpha \right)P_{s}g_{sp}+P_{a}g_{ap}+\sigma_{p}^{2}}. \end{array}$$

Further, to ensure tractability in deriving the CDF expression for $\gamma_{p}$, consider the scenario in $\gamma_{p_{2}}$ where $P_{a}g_{ap}+\sigma_{p}^{2}\ll P_{s}g_{sp}$ (i.e., the secondary power received at the PR is considerably stronger compared to that of the adversary and noise powers). In this case, the expression for $\gamma_{p_{2}}$ can be further simplified to

(4) $$\begin{array}{cc} \gamma_{p_{2}} & =\frac{\alpha }{1-\alpha +\frac{P_{a}g_{ap}+\sigma_{p}^{2}}{P_{s}g_{sp}}}\\ \; & \approx \frac{\alpha }{1-\alpha }. \end{array}$$

For this case, we can approximate $\gamma_{p}$ as

(5) $$\gamma_{p}\approx \gamma_{p_{1}}+\frac{\alpha }{1-\alpha }.$$

The distribution function of $\gamma_{p_{1}}$ can be written as

(6) $$F_{p_{1}}\left(x\right)=1-\exp\left(-\frac{\sigma_{p}^{2}}{\lambda_{pp}P_{p}}x\right)\frac{1}{1+\left(1-\alpha \right)\frac{\lambda_{sp}P_{s}}{\lambda_{pp}P_{p}}x}\frac{1}{1+\frac{\lambda_{ap}P_{a}}{\lambda_{pp}P_{p}}x}.$$

Following the results of (5) and (6), the CDF of $\gamma_{p}$ is calculated using

(7) $$F_{p}\left(x\right)=1-\frac{\exp\left(-\gamma_{np}\left(x-\frac{\alpha }{1-\alpha }\right)\right)}{\left(1+\left(1-\alpha \right)\gamma_{sp}\left(x-\frac{\alpha }{1-\alpha }\right)\right)\left(1+\gamma_{ap}\left(x-\frac{\alpha }{1-\alpha }\right)\right)}$$

where $\gamma_{np}=\frac{\sigma_{p}^{2}}{\lambda_{pp}P_{p}}$, $\gamma_{sp}=\frac{\lambda_{sp}P_{s}}{\lambda_{pp}P_{p}}$, and $\gamma_{ap}=\frac{\lambda_{ap}P_{a}}{\lambda_{pp}P_{p}}$. Let $\rho_{p}$ denote the average outage probability of the received primary IoT signal at the PR; thus, $\rho_{p}$ can be expressed as

(8) $$\begin{array}{cc} \rho_{p} & =P\left\{\log_{2}\left(1+\gamma_{p}\right)\leq R_{p}\right\}\\ \; & =P\left\{\gamma_{p}\leq \theta_{p}\right\}\\ \; & =F_{p}\left(\theta_{p}\right)\\ \; & =1-\frac{\exp\left(-\left(\theta_{p}-\frac{\alpha }{1-\alpha }\right)\gamma_{np}\right)}{\left(1+\left(1-\alpha \right)\left(\theta_{p}-\frac{\alpha }{1-\alpha }\right)\gamma_{sp}\right)\left(1+\left(\theta_{p}-\frac{\alpha }{1-\alpha }\right)\gamma_{ap}\right)} \end{array}$$

where P{·} is the probability operator and $\theta_{p}=2^{R_{p}}-1$.

Similarly, the CDF of the SINR of ST’s signal at its intended receiver SR (i.e., $\gamma_{s}$) can be expressed as

(9) $$F_{s}\left(x\right)=1-\frac{\exp\left(-\gamma_{ns}\frac{x}{1-\alpha }\right)}{\left(1+\gamma_{ps}\frac{x}{1-\alpha }\right)\left(1+\gamma_{as}\frac{x}{1-\alpha }\right)}$$

where $\gamma_{ns}=\frac{\sigma_{s}^{2}}{\lambda_{ss}P_{s}}$, $\gamma_{ps}=\frac{\lambda_{ps}P_{p}}{\lambda_{ss}P_{s}}$, and $\gamma_{as}=\frac{\lambda_{as}P_{a}}{\lambda_{ss}P_{s}}$. Then, the average outage probability of ST’s signal received at its intended receiver unit is found from

(10) $$\begin{array}{cc} \rho_{s}\; & =1-\frac{\exp\left(-\frac{\theta_{s}}{1-\alpha }\gamma_{ns}\right)}{\left(1+\frac{\theta_{s}}{1-\alpha }\gamma_{ps}\right)\left(1+\frac{\theta_{s}}{1-\alpha }\gamma_{as}\right)} \end{array}$$

where $\theta_{s}=2^{R_{s}}-1$.

The development above shows that the $F_{p}$ moves to the right as α increases, as increasing the value of α leads to increasing the $\frac{\alpha }{1-\alpha }$ term in the CDF formula in Equation (7), leading to a shift to the right. Furthermore, the secondary CDF formula in (9) explains the impact of varying the cooperation factor on the $F_{s}$. In addition, when α increases, the primary outage probability decreases while the secondary IoT device’s outage probability increases as indicated in Equations (8) and (10).

### 3.3. Transmission Strategy

Let $N_{s}$ represent the number of secondary devices in the IoT environment. Suppose that $\zeta_{p}$ and $\zeta_{s}$ are the outage levels that the primary IoT device (i.e., PT) and the secondary IoT devices (i.e., ST) can tolerate, respectively. In practice, we have $0<\zeta_{p}\ll \zeta_{s}<1$. To mitigate the negative results of the interference attack on the PR, one secondary device is chosen from the pool of $N_{s}$ IoT devices to cooperate with the PT. To enable cooperation with the PT and to simultaneously transmit its own data, the selected secondary IoT device needs to utilize a cooperation factor $\alpha \leq \alpha_{\max}$ that ensures that the following constraints are satisfied:

(11) $$\begin{array}{cc} \rho_{p} & \leq \zeta_{p}\\ \rho_{s} & \leq \zeta_{s}. \end{array}$$

This formula allows the PT and ST to cooperate to mitigate the impact of the interference attack caused by the AT by limiting the PT’s signal average outage probability to a level of $\zeta_{p}$. This ensures that the PT maintains a certain level of information availability. Simultaneously, the formulation also provides the ST with an opportunity to communicate over the wireless channel while guaranteeing a limited outage probability $\zeta_{s}$ for the ST. This approach offers a balance between ensuring information availability for the PT and enabling limited communication for the ST in the presence of interference.

Consider the case of fixed $P_{s}$ and α values. Let $A_{1}=\frac{\exp\left(-\left(\theta_{p}-\frac{\alpha }{1-\alpha }\right)\gamma_{np}\right)}{1+\left(\theta_{p}-\frac{\alpha }{1-\alpha }\right)\gamma_{ap}}$ and $B_{1}=\left(1-\alpha \right)\left(\theta_{p}-\frac{\alpha }{1-\alpha }\right)$ in (8); then, the value of $\rho_{p}$ can be expressed as

(12) $$\rho_{p}=1-\frac{A_{1}}{1+B_{1}\gamma_{sp}}.$$

Following the transmission constrains in (11), the limit on $\gamma_{sp}$ is rephrased as

(13) $$\gamma_{sp}\leq \frac{A_{1}-1+\zeta_{p}}{\left(1-\zeta_{p}\right)B_{1}}.$$

Similarly, let $A_{2}=\frac{\exp\left(-\frac{\theta_{s}}{1-\alpha }\gamma_{ns}\right)}{1+\frac{\theta_{s}}{1-\alpha }\gamma_{as}}$ and $B_{2}=\frac{\theta_{s}}{1-\alpha }$ in (10); the value of $\rho_{s}$ becomes

(14) $$\rho_{s}=1-\frac{A_{2}}{1+B_{2}\gamma_{ps}}\;.$$

Using the constraint on $\rho_{s}$ in (11) and the development in (14), $\gamma_{ps}$ is limited as

(15) $$\gamma_{ps}\leq \frac{A_{2}-1+\zeta_{s}}{\left(1-\zeta_{s}\right)B_{2}}.$$

Recall that $\gamma_{sp}=\frac{\lambda_{sp}P_{s}}{\lambda_{pp}P_{p}}$ and $\gamma_{ps}=\frac{\lambda_{ps}P_{p}}{\lambda_{ss}P_{s}}$; then, the secondary IoT device has to satisfy the following constraints on the transmission power:

(16) $$\begin{array}{cc} P_{s} & \leq \frac{A_{1}-1+\zeta_{p}}{1-\zeta_{p}}\frac{\lambda_{pp}}{\lambda_{sp}}\frac{P_{p}}{B_{1}}\\ P_{s} & \geq \frac{1-\zeta_{s}}{A_{2}-1+\zeta_{s}}\frac{\lambda_{ps}}{\lambda_{ss}}B_{2}P_{p}. \end{array}$$

The cooperative transmission strategy proposed in this work to satisfy the PT’s information availability requirements is illustrated in Algorithm 1. In the proposed transmission strategy, each secondary IoT device has its own constraints and environment settings, including parameters such as $\zeta_{s}$, $\alpha_{\max}$, $\lambda_{ss}$, $\lambda_{sp}$, $R_{s}$, $P_{s}$, and others. The proposed algorithm verifies each candidate ST in a round-robin fashion to determine if it satisfies the transmission criteria outlined in (11). The algorithm begins by collecting and estimating the communication environment setting parameters, including the number of secondary IoT devices, channel strengths between the devices, noise levels, transmission rates and powers, and outage probability requirements. Each secondary IoT device is then verified to determine if it satisfies the proposed transmission criteria in (11).
**Algorithm 1:** Transmission Strategy for Interference Attacks DefenseDetermine: $\zeta_{p}$.Collect: $P_{p}$, $P_{a}$, $R_{p}$, $\sigma_{p}^{2}$, $\sigma_{s}^{2}$.Estimate: $\lambda_{pp}$, $\lambda_{ps}$, $\lambda_{ap}$, $\lambda_{as}$.Determine: $N_{s}$.**while** TRUE **do**   **if** PT has no more data to transmit **then**     Break.   **end if**   Initialize: n←1.   **while** $n\leq N_{s}$ **do**        Determine: ${\mathrm{ST}}_{n}$.        Determine: $P_{s}$, $R_{s}$, $\lambda_{ss}$, $\lambda_{sp}$ of ${\mathrm{ST}}_{n}$.        Determine: $\zeta_{s}$, $\alpha_{\max}$.        Calculate: $S_{\alpha }\leftarrow \left\{0<\alpha \leq \alpha_{\max}\right\}$ that satisfies outage requirements.        **if** $S_{\alpha }\neq \left[\right]$ **then**          Assign: ST ← ${\mathrm{ST}}_{n}$.          Assign: $\alpha \leftarrow \max\left(S_{\alpha }\right)$.          **while** TRUE **do**             Access: ST uses $\alpha P_{s}$ for PT’s signal and $\left(1-\alpha \right)P_{s}$ for its signal.             **if** ST has no more data to transmit **then**               Break.             **end if**          **end while**        **end if**        n←n+1.   **end while****end while**

During each transmission interval, the scheduled secondary IoT device retransmits the primary signal with a transmission power of $\alpha P_{s}$ while also communicating its own signal with a transmission power of $\left(1-\alpha \right)P_{s}$ using the shared channel. Then, data transmission by the ST alters the SINR value of the PT’s signal that is received at the PR. However, by ensuring that the ST’s transmission satisfies the constraints in (11), the average outage probability of the PT remains below the maximum threshold of $\zeta_{p}$, and the ST experiences an average outage probability less than its limit of $\zeta_{s}$. Even though there is an interference attack by the AT, the information availability constraint is fulfilled for the primary device due to the cooperative secondary communication. Simultaneously, the cooperating secondary device is granted an opportunity to communicate over the shared wireless channel, achieving a less stringent outage probability constraint.

## 4. PLS for Eavesdropping Attacks Defense

The same principles can be employed to devise a PLS collaborative approach to enhance confidentiality against eavesdropping. In this case, we consider a setup with several IoT devices communicating their sensor measurements using a wireless communication channel. A set of the IoT devices, termed as primary devices, require higher signal quality guarantees at the receiver compared with other secondary IoT devices, which have lower transmission priority. Again, the primary and secondary devices may use different receiving units. Additionally, there is an illegitimate device, referred to as the eavesdropper, attempting to decode the primary device’s transmission. We develop a coordinated transmission strategy by secondary IoT devices to ensure the information confidentiality of the primary device’s signal in the presence of the eavesdropper.

When secondary transmissions occur, they introduce interference to the communication system, which can be detected by both the PR and the eavesdropper EVE. Also, primary transmissions will cause interference at the SR. Using a spectrum-sharing communication paradigm, secondary devices transmit with the primary device simultaneously. The simultaneous transmission occurs while ensuring a minimum quality level of the received primary signal, measured by satisfying an average primary outage probability constraint. Further, the simultaneous transmission of the signals adds extra interference to the received signal at the EVE, thus making it more challenging for the EVE to decode the primary signal. This approach helps the primary IoT device achieve a confidentiality level. The PT utilizes the ST secondary transmission to inflict a signal outage at the EVE, again preventing the EVE from decoding the PT’s signal and thus ensuring confidentiality in its transmission.

This innovative transmission scheme enables IoT devices to communicate wirelessly while strategically inducing channel outages to prevent eavesdroppers from decoding the transmitted signals. An algorithmic transmission strategy that enables IoT devices, threatened by an eavesdropper, is developed to collaborate and cause signal outages, thus reducing the eavesdropper’s ability to decode the signal of interest. This strategy leverages a spectrum-sharing communication model to enhance information confidentiality for IoT devices. By strategically inducing signal outages on the eavesdropper, the IoT devices ensure that sensitive information remains protected during wireless communication.

### 4.1. System Model

**Figure 3:**
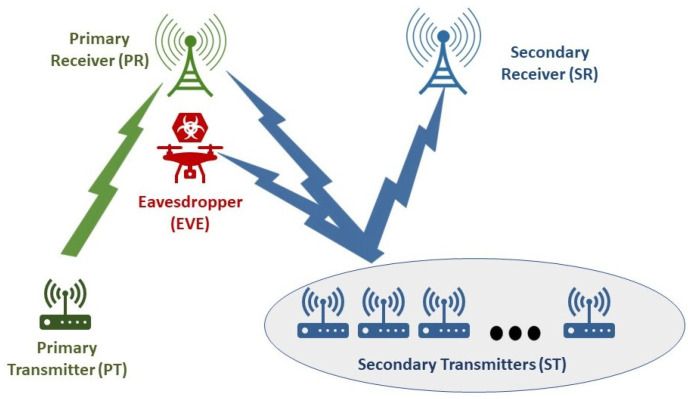
Eavesdropping attacks problem setup.

The wireless communication setup consists of a spectrum-sharing system as shown in Figure 3. This system depicts a primary transmitter communicating with a primary receiver unit using a wireless channel. There also exist multiple secondary transmitters aiming to communicate with another secondary receiver unit. The PR and SR IoT devices can simultaneously transmit their data wirelessly. The threat model considers an adversary, referred to as an EVE, attempting to eavesdrop on data transmitted by the PT. Let the PR transmit at a rate of $R_{p}$ with a power of $P_{p}$; both are assumed to remain constant during the communication period. During every transmission round, a secondary IoT transmitter is chosen to start transmitting with a power of $P_{s}$ over the wireless channel. At the primary receiver, the noise is assumed to be AWGN with a mean of zero and $\sigma_{p}^{2}$ variance. Also, we assume that the eavesdropper EVE and SR have AWGN with respective variances of $\sigma_{e}^{2}$ and $\sigma_{s}^{2}$.

**Figure 4:**
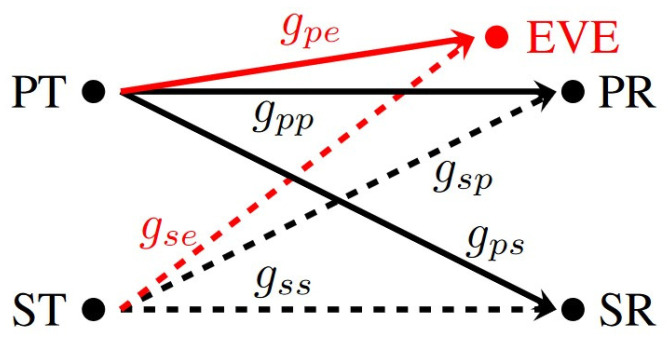
Eavesdropping attacks problem model.

Between the two IoT devices and the receiver units, the wireless channels are modeled as i.i.d. block-fading channels with Rayleigh distribution. Figure 4 illustrates this setup, where the channel power gains between the PT and PR, SR, and EVE are defined as $g_{pp}$, $g_{ps}$, and $g_{pe}$, with corresponding respective averages of $\lambda_{pp}$, $\lambda_{ps}$, and $\lambda_{pe}$. Moreover, channel power gains between the ST and PR, SR, and EVE are defined as $g_{sp}$, $g_{ss}$, and $g_{se}$, with respective averages of $\lambda_{sp}$, $\lambda_{ss}$, and $\lambda_{se}$. Here, the λ’s are different real and positive values that reflect relevant communication environment characteristics.

The cumulative distribution function (CDF) of $g_{pp}$ can be mathematically described as

(17) $$F_{g_{pp}}\left(x\right)=\left(1-\exp\left(-\frac{x}{\lambda_{pp}}\right)\right)u\left(x\right).$$

The CDF mathematical model for other channel power gains such as $g_{ps}$ and $g_{pe}$ will be similar:

(18) $$\begin{array}{cc} F_{g_{ps}}\left(x\right) & =\left(1-\exp\left(-\frac{x}{\lambda_{ps}}\right)\right)u\left(x\right)\\ F_{g_{pe}}\left(x\right) & =\left(1-\exp\left(-\frac{x}{\lambda_{pe}}\right)\right)u\left(x\right). \end{array}$$

### 4.2. Cooperation Model

Let $\gamma_{e}$ and $\gamma_{p}$ denote the SINR of the PT’s signal at the EVE and at the PR, respectively, and let the SINR of the ST’s signal at its own receiver unit (i.e., SR) be termed as $\gamma_{s}$. Then, with concurrent transmissions from the primary and secondary, the previous SINR values can be expressed as

(19) $$\begin{array}{cc} \gamma_{p} & =\frac{g_{pp}P_{p}}{g_{sp}P_{s}+\sigma_{p}^{2}}.\\ \gamma_{e} & =\frac{g_{pe}P_{p}}{g_{se}P_{s}+\sigma_{e}^{2}}.\\ \gamma_{s} & =\frac{g_{ss}P_{s}}{g_{ps}P_{p}+\sigma_{s}^{2}}. \end{array}$$

Further, the CDF cof $\gamma_{p}$ an be calculated using

(20) $$\begin{array}{cc} F_{p}\left(x\right) & =P\left\{\gamma_{p}\leq x\right\}\\ \; & =P\left\{\frac{g_{pp}\;P_{p}/\sigma_{p}^{2}}{g_{sp}\;P_{s}/\sigma_{p}^{2}+1}\leq x\right\}\\ \; & =P\left\{g_{pp}\leq \frac{x}{P_{p}/\sigma_{p}^{2}}\left(g_{sp}\;P_{s}/\sigma_{p}^{2}+1\right)\right\}\\ \; & =\int_{0}^{\infty }\left(1-\exp\left(-\frac{x(yP_{s}/\sigma_{p}^{2}+1)}{\lambda_{pp}P_{p}/\sigma_{p}^{2}}\right)\right)\frac{\exp\left(-\frac{y}{\lambda_{sp}}\right)}{\lambda_{sp}}dy. \end{array}$$

This integration is simplified as

(21) $$F_{p}\left(x\right)=\left(1-\frac{\exp\left(-\frac{x}{\lambda_{pp}P_{p}/\sigma_{p}^{2}}\right)}{1+\frac{\lambda_{sp}}{\lambda_{pp}}\frac{P_{s}}{P_{p}}x}\right)u\left(x\right).$$

Following a similar derivation process for $\gamma_{e}$ CDF results in

(22) $$F_{e}\left(x\right)=\left(1-\frac{\exp\left(-\frac{x}{\lambda_{pe}P_{p}/\sigma_{e}^{2}}\right)}{1+\frac{\lambda_{se}}{\lambda_{pe}}\frac{P_{s}}{P_{p}}x}\right)u\left(x\right).$$

An outage in the wireless communication channel happens when the transmitted data rate exceeds the capacity of the channel. Hence, the outage probability of the PT’s transmission when measured at the PR can be expressed using $\rho_{p}=P\left\{\log_{2}\left(1+\gamma_{p}\right)\leq R_{p}\right\}=P\left\{\gamma_{p}\leq 2^{R_{p}}-1\right\}$. With (21), this leads to an outage probability of the PT as

(23) $$\rho_{p}=1-\frac{\exp\left(-\frac{2^{R_{p}}-1}{\lambda_{pp}P_{p}/\sigma_{p}^{2}}\right)}{1+\frac{\lambda_{sp}}{\lambda_{pp}}\frac{P_{s}}{P_{p}}\left(2^{R_{p}}-1\right)}.$$

Following a similar derivation, the average channel outage probability of the EVE is expressed as $\rho_{e}=P\left\{\log_{2}\left(1+\gamma_{e}\right)\leq R_{p}\right\}$. With the results in (22), the outage probability is found to be

(24) $$\rho_{e}=1-\frac{\exp\left(-\frac{2^{R_{p}}-1}{\lambda_{pe}P_{p}/\sigma_{e}^{2}}\right)}{1+\frac{\lambda_{se}}{\lambda_{pe}}\frac{P_{s}}{P_{p}}\left(2^{R_{p}}-1\right)}.$$

In a spectrum-sharing communication system, a secondary transmission could be controlled by limiting the additional interference that is received at the primary receiver unit. In the described setup, the outage probability of the primary signal at the PR is limited with a maximum value of $\zeta_{p}$. This limiting helps to account for the secondary interference such that $\rho_{p}\leq \zeta_{p}$. Hence, the transmission power of the ST is limited to

(25) $$P_{s}\leq \frac{\exp\left(-\frac{2^{R_{p}}-1}{\lambda_{pp}P_{p}/\sigma_{p}^{2}}\right)+\zeta_{p}-1}{\frac{\lambda_{sp}}{\lambda_{pp}}\frac{2^{R_{p}}-1}{P_{p}}\left(1-\zeta_{p}\right)}.$$

Further, the secondary transmission is employed to control the lower limit of the average outage probability of the EVE as $\rho_{e}\geq \zeta_{e}$. Here, $\zeta_{e}\gg \zeta_{p}$, which consequently limits the transmission power of the secondary as

(26) $$P_{s}\geq \frac{\exp\left(-\frac{2^{R_{p}}-1}{\lambda_{pe}P_{p}/\sigma_{e}^{2}}\right)+\zeta_{e}-1}{\frac{\lambda_{se}}{\lambda_{pe}}\frac{2^{R_{p}}-1}{P_{p}}\left(1-\zeta_{e}\right)}.$$

Thus, a level of confidentiality of the PT’s signal at the EVE can be achieved by requiring the transmission power of the secondary to satisfy (25) and (26). By satisfying (25), the ST avoids causing excessive channel outage at the primary receiver, and by satisfying (26), the ST causes more outages at the EVE. The PT’s objective is to transmit its data to the PR while hindering the EVE’s ability to decode the transmitted information. Using the proposed strategy, the PT allows the ST to transmit data over the wireless channel, causing a secondary interference that will results in an additional outage at the PR and EVE. The secondary transmission is controlled such that it causes a lower-limit outage of $\zeta_{e}$ at the EVE and an upper-limit outage of $\zeta_{p}$ at the PR.

### 4.3. Transmission Strategy

To establish the base case before developing the cooperative transmission strategy, consider the case with no secondary transmission (i.e., $P_{s}=0$). Hence,

(27) $$\begin{array}{cc} \gamma_{p_{0}} & =\frac{g_{pp}P_{p}}{\sigma_{p}^{2}}\\ \gamma_{e_{0}} & =\frac{g_{pe}P_{p}}{\sigma_{e}^{2}}. \end{array}$$

The CDF expressions of $\gamma_{p_{0}}$ and $\gamma_{e_{0}}$ will then simplify to

(28) $$\begin{array}{cc} F_{p_{0}}\left(x\right) & =\left(1-\exp\left(-\frac{x}{\lambda_{pp}P_{p}/\sigma_{p}^{2}}\right)\right)u\left(x\right).\\ F_{e_{0}}\left(x\right) & =\left(1-\exp\left(-\frac{x}{\lambda_{pe}P_{p}/\sigma_{e}^{2}}\right)\right)u\left(x\right). \end{array}$$

Then, the outage probability can be evaluated as

(29) $$\begin{array}{cc} \rho_{p_{0}} & =1-\exp\left(-\frac{2^{R_{p}}-1}{\lambda_{pp}P_{p}/\sigma_{p}^{2}}\right).\\ \rho_{e_{0}} & =1-\exp\left(-\frac{2^{R_{p}}-1}{\lambda_{pe}P_{p}/\sigma_{e}^{2}}\right). \end{array}$$

Note here that the symbol subscript of zero in (27)–(29) signifies that $P_{s}=0$ and results in base case values.

Let $P_{s_{L}}$ and $P_{s_{U}}$ designate the lower and upper limits on the secondary transmission power. Then, combining (25), (26), and (29) will result in a set of requirements for transmission power expressed as

(30) $$\begin{array}{cc} P_{s} & \leq P_{s_{U}}=\frac{\zeta_{p}-\rho_{p_{0}}}{1-\zeta_{p}}\frac{\lambda_{pp}}{\lambda_{sp}}\frac{P_{p}}{2^{R_{p}}-1}.\\ P_{s} & \geq P_{s_{L}}=\frac{\zeta_{e}-\rho_{e_{0}}}{1-\zeta_{e}}\frac{\lambda_{pe}}{\lambda_{se}}\frac{P_{p}}{2^{R_{p}}-1}. \end{array}$$

To ensure concurrent transmission over the wireless channel, any secondary transmitter must operate within a specific power range, defined as $P_{s_{L}}\leq P_{s}\leq P_{s_{U}}$. This constraint guarantees that the EVE experiences an outage probability exceeding the minimum requirement ($\zeta_{e}$) while simultaneously ensuring that the primary receiver’s outage probability remains below the maximum threshold ($\zeta_{p}$), where $\zeta_{p}\ll \zeta_{e}$.

The communication system is assumed to be composed of $N_{s}$ available secondary transmitters, each characterized by its unique maximum transmit power ($P_{s_{\max}}$) and channel strength. A round-robin approach is employed to verify if each secondary transmitter can meet the condition in (30). Upon satisfying this criterion, a secondary transmitter is permitted to transmit using a power level of $P_{s}=\min\left(P_{s_{U}},P_{s_{\max}}\right)$. This carefully selected transmission power ensures that the ST adheres to the outage probability requirements for both the EVE and PR.

The transmission strategy depicted in Algorithm 2 outlines the transmission strategy designed to meet the confidentiality constraint. It begins by gathering system parameters, including outage requirements, data rates, noise powers, channel strengths, and the number of potential secondary transmitters. Using a round-robin approach, each secondary transmitter is evaluated to determine if it meets the proposed transmission criteria. If a secondary transmitter satisfies these criteria, it is selected to transmit its data over the shared channel, thereby introducing interference and additional outage to both the EVE and PT. Given that (30) is satisfied for the selected secondary transmitter, the outage probability for the PT will remain within the acceptable limit ($\zeta_{p}$), while the EVE will experience an outage probability of no less than $\zeta_{e}$. As a result, the confidentiality metric is upheld.

**Algorithm 2:** Transmission Strategy for Eavesdropping Attacks Defense
Determine: $\zeta_{p},\zeta_{e}$.Collect: $P_{p}$, $R_{p}$, $\sigma_{p}^{2}$, $\sigma_{e}^{2}$, $\lambda_{pp}$, $\lambda_{pe}$.Calculate: $\rho_{p_{0}}$, $\rho_{e_{0}}$.Determine: $N_{s}$.**while** TRUE **do**   **if** PT has no more data to transmit **then**     Break Loop.   **end if**   Initialize: n←1.   **while** $n\leq N_{s}$ **do**        Determine: ${\mathrm{ST}}_{n}$.        Determine: $\lambda_{sp}$, $\lambda_{se}$ of ${\mathrm{ST}}_{n}$.        Find: $P_{s_{\max}}$.        Calculate: $P_{s_{L}}$, $P_{s_{U}}$.        **if** $P_{s_{L}}\leq P_{s_{U}}$ AND $P_{s_{L}}\leq P_{s_{\max}}$ **then**          Assign: ST ← ${\mathrm{ST}}_{n}$.          Assign: $P_{s}\leftarrow \min\left(P_{s_{U}},P_{s_{\max}}\right)$.          **while** TRUE **do**             Access: ST transmits data with $P_{s}$.             **if** ST has no more data to transmit **then**               Break.             **end if**          **end while**        **end if**        n←n+1.   **end while**
**end while**



Consider the case where the ST communicates over the channel with a rate of $R_{s}$. Given the value of $\gamma_{s}$ in (19), and following a similar development to that of the PT, the CDF of $\gamma_{s}$, termed as $F_{s}$, is calculated using

(31) $$\begin{array}{cc} F_{s}\left(x\right) & =P\left\{\gamma_{s}\leq x\right\}\\ \; & =P\left\{\frac{g_{ss}P_{s}}{g_{ps}P_{p}+\sigma_{s}^{2}}\leq x\right\}\\ \; & =P\left\{g_{ss}\leq \frac{x}{P_{s}/\sigma_{s}^{2}}\left(g_{ps}\;P_{p}/\sigma_{s}^{2}+1\right)\right\}\\ \; & =\int_{0}^{\infty }\left(1-\exp\left(-\frac{x(yP_{p}/\sigma_{s}^{2}+1)}{\lambda_{ss}P_{s}/\sigma_{s}^{2}}\right)\right)\frac{\exp\left(-\frac{y}{\lambda_{ps}}\right)}{\lambda_{ps}}dy. \end{array}$$

This leads to $F_{s}$ being expressed as

(32) $$F_{s}\left(x\right)=\left(1-\frac{\exp\left(-\frac{x}{\lambda_{ss}P_{s}/\sigma_{s}^{2}}\right)}{1+\frac{\lambda_{ps}}{\lambda_{ss}}\frac{P_{p}}{P_{s}}x}\right)u\left(x\right).$$

Next, for a transmission rate of $R_{s}$, the outage probability of the ST’s transmission at the SR is calculated using $\rho_{s}=P\left\{\gamma_{s}\leq 2^{R_{s}}-1\right\}$; then, using $F_{s}$ from (32), the outage probability of the ST becomes

(33) $$\rho_{s}=1-\frac{\exp\left(-\frac{2^{R_{s}}-1}{\lambda_{ss}P_{s}/\sigma_{s}^{2}}\right)}{1+\frac{\lambda_{ps}}{\lambda_{ss}}\frac{P_{p}}{P_{s}}\left(2^{R_{s}}-1\right)}.$$

Recall that the ST has to satisfy the outage probability constraints on the PR and EVE; this means that the ST has upper and lower transmission power limits of $P_{s_{U}}$ and $P_{s_{L}}$, respectively, as indicated in (30). As the ST will try to maximize its received signal level at the SR, $P_{s}=\min\left(P_{s_{U}},P_{s_{\max}}\right)$ as mentioned previously. Given these transmission limits on $P_{s}$, the outage probability of the ST will be bounded as $\rho_{s_{L}}\leq \rho_{s}\leq \rho_{s_{U}}$, where

(34) $$\begin{array}{c} \rho_{s_{L}}=1-\frac{\exp\left(-\frac{2^{R_{s}}-1}{\lambda_{ss}P_{s_{U}}/\sigma_{s}^{2}}\right)}{1+\frac{\lambda_{ps}}{\lambda_{ss}}\frac{P_{p}}{P_{s_{U}}}\left(2^{R_{s}}-1\right)}.\\ \rho_{s_{U}}=1-\frac{\exp\left(-\frac{2^{R_{s}}-1}{\lambda_{ss}P_{s_{L}}/\sigma_{s}^{2}}\right)}{1+\frac{\lambda_{ps}}{\lambda_{ss}}\frac{P_{p}}{P_{s_{L}}}\left(2^{R_{s}}-1\right)}. \end{array}$$

## 5. Results and Discussion

### 5.1. PLS for Interference Attacks Defense

**Figure 5:**
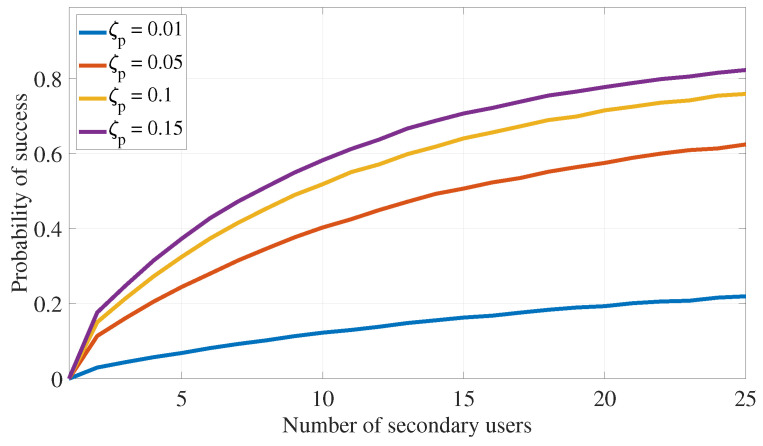
PLS for interference attack defense: impact of the outage constraints on the algorithm success probability ($\zeta_{p}$).

**Figure 6:**
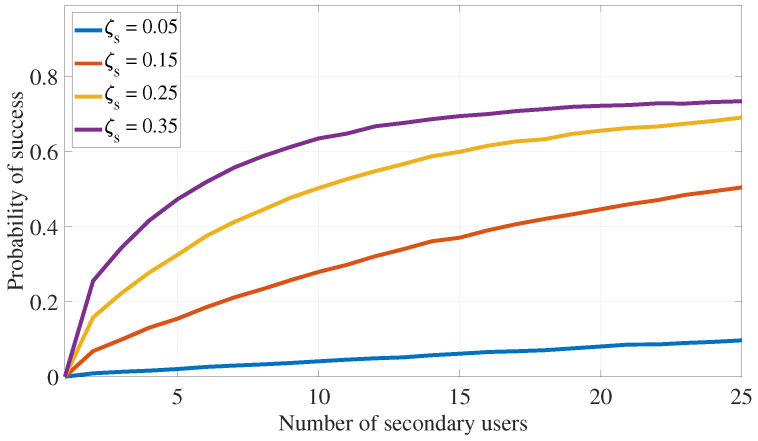
PLS for interference attack defense: impact of the outage constraints on the algorithm success probability ($\zeta_{s}$).

**Figure 7:**
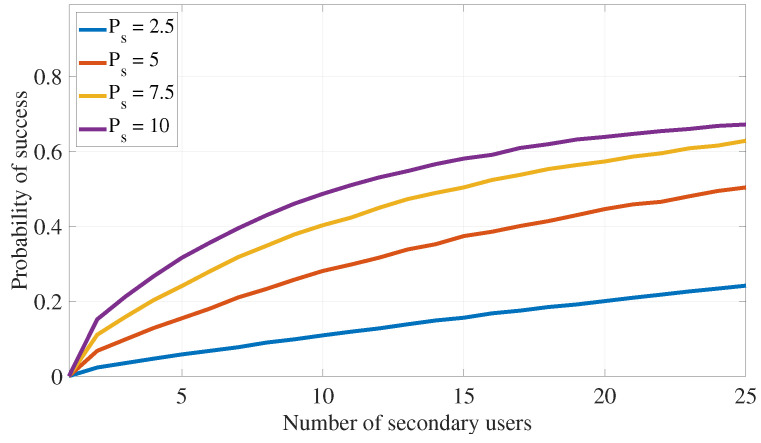
PLS for interference attack defense: impact of the secondary transmission power on the algorithm success probability.

**Figure 8:**
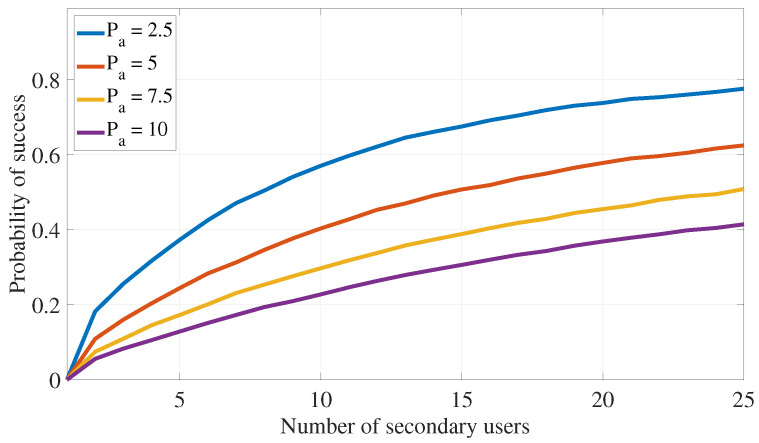
PLS for interference attack defense: impact of $P_{a}$ on the algorithm success probability.

This section assesses the effectiveness of the proposed PLS cooperative transmission scheme outlined in Algorithm 1 for interference attacks defense by demonstrating the rate of finding appropriate secondary devices that satisfy the constraints specified in (11) under different system settings. The following numerical values are used in this section: $\alpha_{\max}=0.49$, $\lambda_{as}=0.75$, $\lambda_{ap}=0.75$, $\lambda_{ss}=1$, $\lambda_{sp}=0.75$, $\lambda_{ps}=0.75$, $\lambda_{pp}=1$, $\sigma_{s}^{2}=0.1$, $\sigma_{p}^{2}=0.1$, $R_{s}=0.5$, $R_{p}=1$, $P_{a}=5$, $P_{s}=7.5$, and $P_{p}=10$. Further, $\zeta_{p}=0.05$ and $\zeta_{s}=0.2$ are also used in Figure 5, Figure 6, Figure 7 and Figure 8.

Recall that the success rate of the proposed transmission strategy can be measured using the probability of selecting appropriate secondary devices that satisfy the transmission constraints outlined in (11). Figure 5 and Figure 6 investigate how the number of available secondary devices ($N_{s}$) impacts the success rate of the communication strategy in Algorithm 1. Here, Figure 5 shows that, with increasing the number of available secondary IoT devices ($N_{s}$), the proposed transmission algorithm has better chances of identifying a secondary device that satisfies the primary and secondary outage probability constraints of (11). Also, this figure confirms that as outage probability constraints ($\zeta_{p}$ or $\zeta_{s}$) become more relaxed (i.e., increase), the proposed transmission algorithm has more chances of identifying secondary IoT devices that satisfy the outage probability constraints of (11), leading the algorithm to achieve higher rates of success.

Finally, Figure 7 and Figure 8 illustrate the impact of varying the amount of transmission power for the secondary user and the adversary, respectively, on the probability of finding a suitable ST that meets the transmission and interference constraints in (11). As expected, increasing available $P_{s}$ enhances the algorithm’s ability to find STs that satisfy the outage probability requirements. On the other hand, higher transmission power for the adversary reduces the algorithm’s success rate.

### 5.2. PLS for Eavesdropping Attack Defense

This section evaluates the efficacy of the proposed PLS cooperative transmission algorithm, as illustrated in Figure 3, in defending against eavesdropping attacks. Through simulation results, we demonstrate that the transmission strategy presented in Algorithm 2 successfully achieves the target outage probability requirements for both the EVE and the primary receiver. The numerical analysis in this section focuses on the probability of identifying suitable secondary transmitters that satisfy the conditions specified in (30) under various system configurations. For the subsequent numerical results, we assume the following parameters: primary transmitter power $P_{p}=1$, primary transmission rate $R_{p}=1$, and noise power $\sigma^{2}=0.1$ at the PR, EVE, and SR. Also, let $\lambda_{sp}=0.75$, $\lambda_{se}=0.5$, $\lambda_{ss}=1$, $\lambda_{pp}=1$, $\lambda_{pe}=0.5$, and $\lambda_{ps}=0.75$.

**Figure 9:**
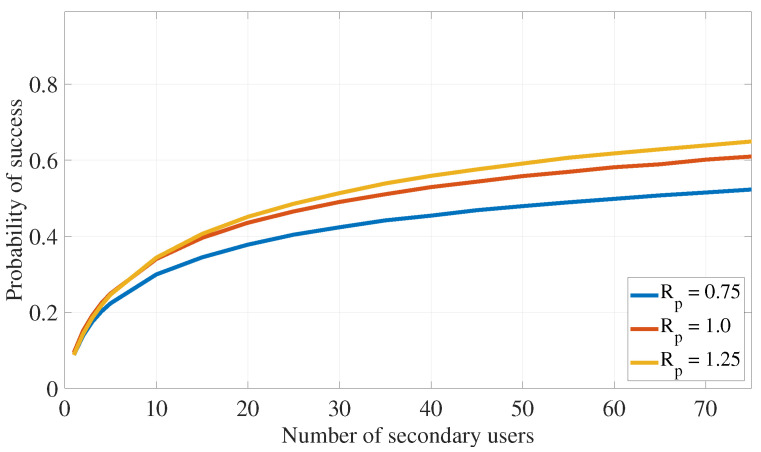
PLS for eavesdropping attack defense: impact of number of transmitters.

Figure 9 examines how the number of available secondary transmitters affects the algorithm’s success rate. In this analysis, the secondary transmission power is constrained between $P_{s_{\min}}=0.75\times P_{p}$ and $P_{s_{\max}}=1.25\times P_{p}$. The target outage probabilities are set at $\zeta_{e}=0.8$ for the EVE and $\zeta_{p}=0.2$ for the PR. As $N_{s}$ increases, the likelihood of identifying a secondary transmitter that satisfies the conditions in (30) also rises. Additionally, higher primary transmission rates, combined with secondary interference, make it more challenging for the EVE to successfully decode the primary signal. This results in increased outages at the EVE and, consequently, a higher probability of finding suitable secondary transmitters.

**Figure 10:**
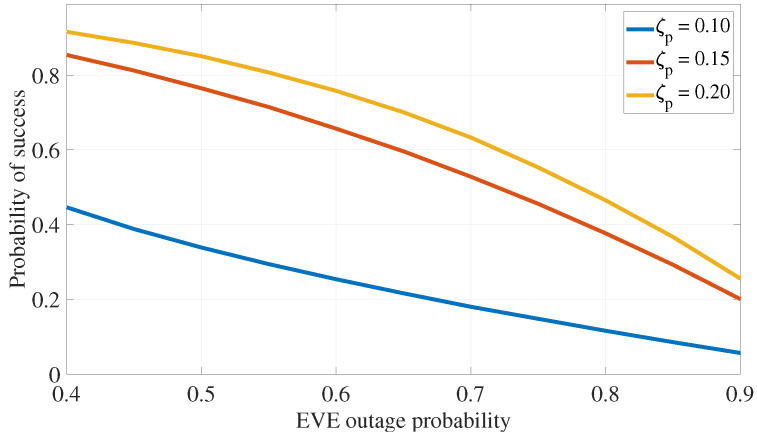
PLS for eavesdropping attack defense: impact of outage requirement.

The impact of varying outage probability requirements at the EVE and the primary receiver is examined, with the number of secondary transmitters set to $N_{s}=25$ and power limits of $P_{s_{\max}}=1.25\times P_{p}$ and $P_{s_{\min}}=0.75\times P_{p}$. The results show that relaxing the outage requirements, either by increasing the acceptable primary outage or reducing the EVE’s outage probability, leads to higher success rates, as illustrated in Figure 10.

**Figure 11:**
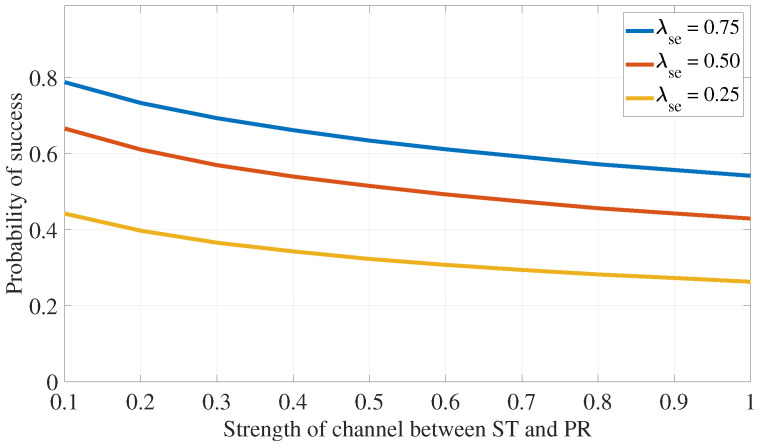
PLS for eavesdropping attack defense: impact of secondary channel strength.

**Figure 12:**
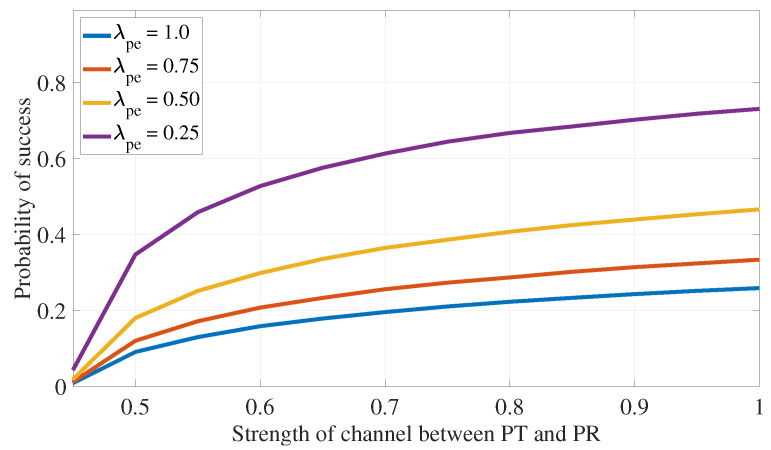
PLS for eavesdropping attack defense: impact of primary channel strength.

The impact of channel strength is investigated, revealing that an increase in $\lambda_{sp}$ results in lower success rates due to more stringent transmission limits for the ST. Conversely, higher values of $\lambda_{pp}$ improve success probability by allowing the ST to transmit at lower power levels. Similar trends are observed for the effects of increasing $\lambda_{se}$ and $\lambda_{pe}$ on the algorithm’s success rate. These observations are illustrated in Figure 11 and Figure 12.

**Figure 13:**
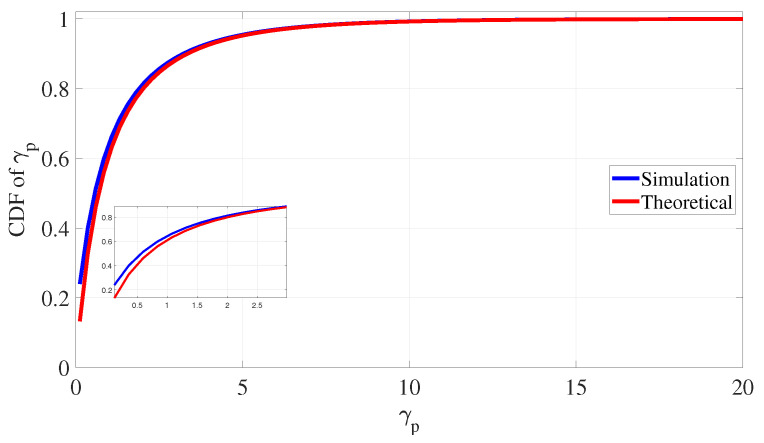
PLS for eavesdropping attack defense: simulated and theoretical CDF of $\gamma_{p}$.

Figure 13 compares the simulated and theoretical values of the CDF of $\gamma_{p}$, where the values of $P_{p}=P_{s}=\lambda_{pp}=\lambda_{sp}=1$ and $\sigma_{p}^{2}=0.1$ are used in calculating the theoretical value in (21) and simulating the environment. The figure confirms that the simulated CDF is very close to the theoretical one, with a very small gap for very low SINR values.

For the next three figures, consider a simulated communication environment similar to the one shown in Figure 4. Let $N_{s}=100$ with a transmission rate of $R_{s}=0.5$ bit/sec/Hz. The PT transmits at a rate of $R_{p}=1$ bit/sec/Hz with $P_{p}=1$ power unit. Similarly, $\sigma^{2}=0.01$ power unit at the PR, SR, and EVE. Let also $\lambda_{sp}=0.5$, $\lambda_{se}=0.75$, $\lambda_{ss}=1$, $\lambda_{pp}=1$, $\lambda_{pe}=0.75$, and $\lambda_{ps}=0.5$. Further, assume that $\zeta_{p}=0.05$ as the maximum primary outage requirement and $\zeta_{e}=0.85$ as the minimum eavesdropper outage probability requirement. Let $P_{s_{\max}}=10P_{p}$ and $P_{s_{\min}}=0.95P_{p}$. For a representative simulated communication environment with 1000 trials, each has 100 block-fading periods.

**Figure 14:**
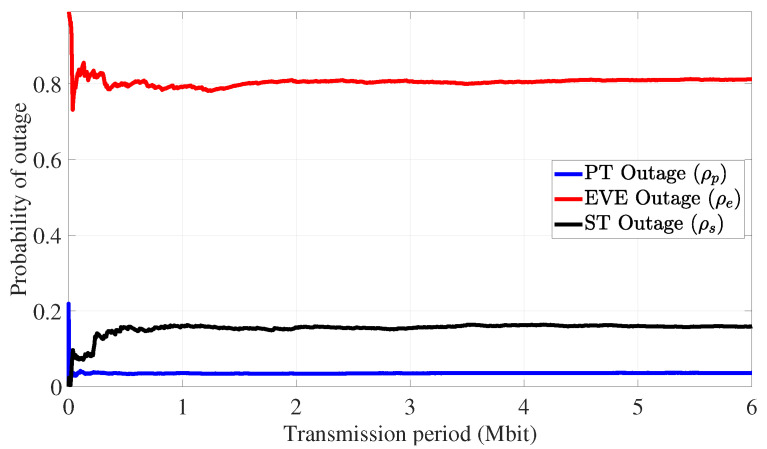
PLS for eavesdropping attack defense: moving average of outage probability over time.

**Figure 15:**
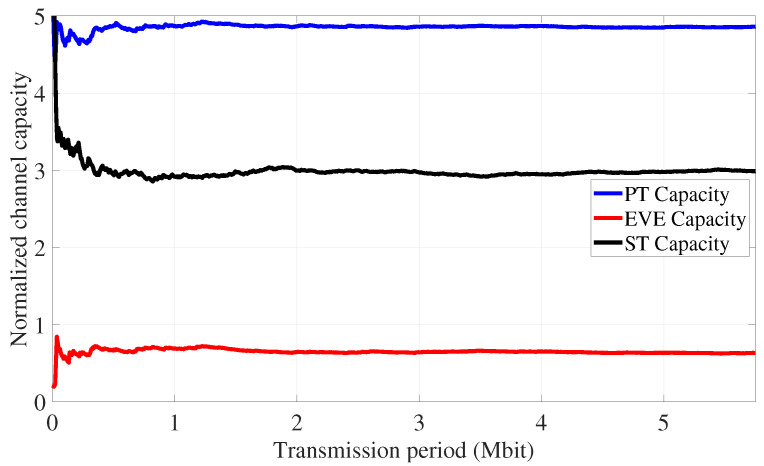
PLS for eavesdropping attack defense: moving average of channel capacity over time.

Figure 14 illustrates the outage probabilities experienced at the PR, EVE, and SR (i.e., $\rho_{p}$, $\rho_{e}$, and $\rho_{s}$) following the implementation of the proposed coordinated transmission strategy in Algorithm 2, and Figure 15 displays the channel capacity of users in the IoT environment. As shown in Figure 14, the achieved outage probability at the PR and EVE are about 5% and 85%, respectively, as predicted by (30) and in Algorithm 2. In addition, the results of Figure 15 emphasize the diminished channel conditions that the eavesdropper experiences compared to the primary and secondary IoT devices.

**Figure 16:**
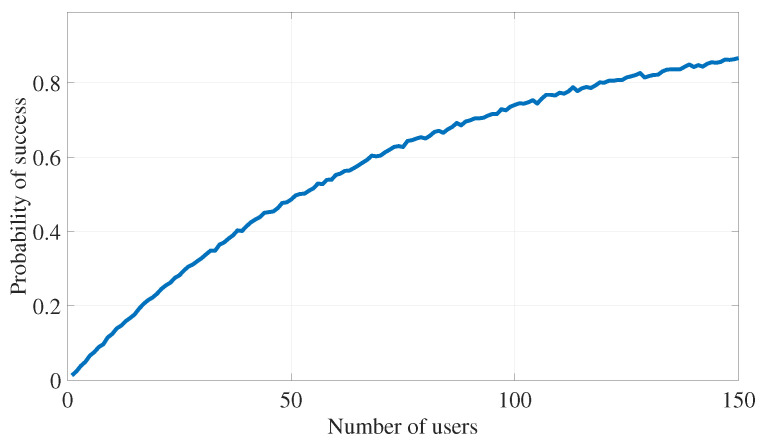
PLS for eavesdropping attack defense: algorithm’s success rate versus number of users.

In addition, the results of Figure 16 show the probability that the cooperative transmission strategy of Algorithm 2 is successful in finding users that help to mitigate the eavesdropping attack on the PT. The figure confirms our intuition that the transmission strategy is more likely to find suitable users that achieve the target outage probability requirements for both the EVE and PR while increasing the pool of available users to choose from.

### 5.3. Discussions

General observations from the above numerical results include that there is a better chance of mitigating interference attacks with an increasing number of IoT devices; this result favors large-scale IoT environments. Further, relaxing the information availability constraints for the primary and/or secondary IoT devices (through having higher outage probability constraints) leads to better success rates in finding suitable STs that could counter the interference attack. Additionally, the algorithm has a better success rate with higher secondary transmission power and/or lower adversary interference power. The numerical results demonstrate the feasibility in using the proposed cooperative IoT transmission strategy in Algorithm 1 to combat interference attacks and maintain information availability. Also, the performance metrics and the practical advantages of using this strategy are supported by the analytical discussions in Section 4.3.

The proposed transmission strategy only relies on the knowledge of the channel gains between the IoT devices, receiver units, and the eavesdropper. Presented numerical results illustrate the proposed algorithm practicality and the capability of IoT devices to concurrently meet the desired signal quality and availability and confidentiality objectives. This approach demonstrates that, by leveraging spectrum-sharing and collaborative transmission strategies, IoT devices can effectively protect sensitive information while maintaining efficient communication performance in wireless environments.

While recent research on physical-layer security is advancing, the focus has primarily been on information-theoretic solutions, with practical implementations being less common. This work proposes algorithmic transmission strategies to achieve uplink IoT information integrity and confidentiality in the presence of adversaries. The proposed solution is tailored to IoT systems, considering the computational and energy limitations of IoT devices by restricting the number of retransmissions and necessary information for the algorithmic transmission strategy. Moreover, the solution accommodates IoT environments by allocating transmission opportunities to available IoT devices based on their channel strengths. The approach also incorporates elements from spectrum-sharing systems to facilitate device cooperation and concurrent transmissions.

## 6. Conclusions

A cooperative IoT transmission strategy is presented in this article to enhance information security in IoT environments; specifically, this work focuses on ensuring IoT information availability during jamming interference attacks and ensuring IoT information confidentiality during eavesdropping attacks. This research contributes to tackling security challenges inherent in wirelessly connected IoT devices and emphasizes the importance of safeguarding information availability and confidentiality across diverse IoT applications and critical industrial processes.

The proposed PLS algorithm for interference attack defense facilitates cooperative communication among IoT devices by involving secondary devices, aiming to maintain the desired outage probability for the primary device and achieve a certain level of information availability. Through relaying the primary device’s data, secondary devices actively contribute and help to meet the primary device’s outage probability requirements. The numerical results presented in this article demonstrate the effectiveness of the proposed transmission strategy, particularly in large-scale IoT environments. The findings emphasize that, by applying the proposed solution, the IoT devices have the capability to attain specific levels of information security even when facing interference attacks. The proposed PLS algorithmic transmission strategy for eavesdropping attack defense employs secondary IoT devices to ensure the quality of IoT signals while deliberately causing channel outages that hinder eavesdroppers from decoding the IoT transmission effectively. Through this collaborative transmission strategy, eavesdroppers’ capability to intercept and decipher sensitive IoT signals is significantly restricted.

## Data Availability

Data are available upon request.
